# Surgical palliative care: A multidisciplinary perspective on where we stand and how we need to evolve

**DOI:** 10.1371/journal.pmed.1005188

**Published:** 2026-08-04

**Authors:** Samuel M. Miller, Paige DeGoursey, Kevin G. Billingsley

**Affiliations:** 1 Department of Surgery, Yale School of Medicine, New Haven, Connecticut, United States of America; 2 Department of Nursing, Yale-New Haven Hospital, New Haven, Connecticut, United States of America

## Abstract

From the perspectives of a bedside nurse, surgical critical care fellow and surgical oncologist, this Perspective by Samuel Miller, Paige DeGoursey and Kevin Billingsley discusses how and when surgical palliative care (SPC) can be best practiced, highlighting different models and advocating for SPC becoming the standard of practice.

## Teaser

Surgical palliative care (SPC) integrates the principles of symptom-based care and relief of suffering into traditional surgical practice. From the perspectives of a bedside nurse, surgical critical care fellow, and surgical oncologist, there are natural opportunities to incorporate these principles and in doing so, progress towards SPC becoming the standard of practice.

## Introduction

Palliative care seeks to improve quality of life for patients with serious illnesses. The integration of these principles into surgical care first appears in medical literature in the early 2000s as ‘surgical palliative care’ (SPC) [1]. Unfortunately, surgical patients are still less likely to receive palliative care consults than patients admitted to medical teams [2,3]. In this Perspective, we join the voices of a bedside nurse, a surgical critical care fellow, and an attending surgical oncologist to define SPC, describe delivery models, identify practice opportunities, and recognize barriers to integration. In this way, we advocate for SPC becoming the standard of care for surgical patients.

## What is surgical palliative care?

SPC has evolved to address a diverse range of healthcare needs. These include providing management for symptoms, pain, and suffering, transitioning to comfort-based care based on patient preferences and clinical judgement, and effectively communicating with patients and families about prognoses, advance directives, and end-of-life decisions [4]. SPC can also include procedures performed with palliative intent [5]: a venting gastrostomy tube can lessen the nausea experienced with malignant bowel obstruction [6]; spinal stabilization can decrease pain from spinal metastases [7]. Meanwhile, nonoperative SPC can involve adjusting to new functional or cognitive baselines after unexpected health events as well as post-hospital discharge planning [8]. SPC facilitates transparent communication about how procedural interventions don’t always align with a patient’s goals.

## Models for delivery of SPC

**Fig 1:**
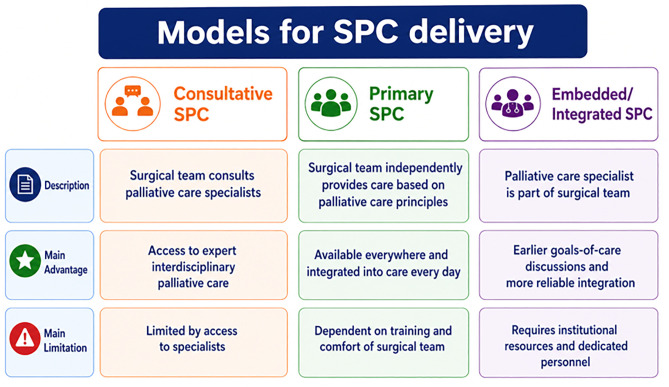
Models for surgical palliative care delivery.

There are multiple models for SPC delivery, several of which are described in Fig 1*.* The optimal model depends on resource availability. With the ongoing shortage of specialized palliative care providers, primary palliative care or palliative care offered by clinicians without fellowship training, are often the models of choice [9]. Implementation efforts should be overseen by a provider with fellowship training in palliative care or who has significant prior experience with the practice of palliative medicine. The successful delivery of SPC ultimately requires training for all members of the care team, including surgeons, nurses, trainees, case managers, pharmacists, social workers, and others.

## Opportunities to practice surgical palliative care

Along a treatment course, there are natural opportunities to practice primary SPC for every member of the healthcare team. The initial consideration of whether to offer surgery is generally the first such opportunity for the surgeon. The surgeon steps back from technical details and considers the bigger picture—*can* surgery help my patient? *Will* surgery help my patient live a life of value? If surgery isn’t aligned with my patient’s goals, *how* do I help them understand it is okay to choose a nonoperative approach?

If the surgeon decides to offer surgery, an informed consent discussion follows. The surgeon describes the possible treatment options, both surgical and nonsurgical, along with the potential risks and benefits of each. This discussion should include reflection on the patient’s prognosis, what the patient hopes surgery will accomplish, and what matters most to them moving forward. For a healthy patient with appendicitis, this conversation may be quite short. But for a patient with metastatic pancreatic cancer and recurrent bowel obstruction, this discussion will be quite different. Once pancreatic cancer is metastatic, prognosis is poor and surgery has not been shown to improve outcomes. If the patient values time at home with family, a venting gastrostomy tube along with hospice care at home may be more aligned with the patient’s goals than a bowel resection or bypass. In these situations, the surgeon can help the patient and family understand that more treatment is not necessarily the path to better quality of life.

Discussion of perioperative code status should include advance directives, which provide the patient’s instructions for medical care in the case that they cannot communicate their own wishes (e.g., due to illness or incapacity). For patients who have never considered a healthcare proxy or living will, this can be the impetus to have important conversations with loved ones and complete paperwork [10]. For those who have established advance directives, this is a chance to confirm their wishes and discuss unique considerations in the perioperative space. This discussion should cover patient preference for care in the event of clinical decline and establish a timeline to revisit goals of care if postoperative recovery is not progressing well. These discussions facilitate the surgical team caring for the patient in accordance with their values and can ease the burden of decision-making on the family.

Surgical trainees encounter opportunities for SPC practice as well. Surgical patients often present with symptoms that have become unmanageable. These symptoms are treated as the patient is worked up and the underlying pathology is identified. Once the pathology becomes clear, many surgeons will unknowingly shift their focus to how they can fix the problem and may lose sight of their patient’s suffering [9]. This is often the beginning of divergence between the surgeon’s and patient’s ideas of what constitutes successful treatment. In these moments, trainees can maintain the surgical team’s connection to the patient and their symptoms. Trainees can focus on ‘symptoms’ as a separate section in their presentations to the team, confirming that the whole team is aware of what the patient is experiencing and how their symptoms have progressed. Surgical trainees can be the glue that keeps patient and surgeon goals aligned.

Bedside nurses are an invaluable part of SPC delivery. As the team member most often at the bedside, a nurse’s insight is unique. Through observation and direct assessment, nurses are able to identify the most distressing factors affecting both the patient and their family [11]. This, alongside the clinical rapport developed between the nurse and their care-recipients, allows nurses to provide a form of highly-individualized patient advocacy. This can take many forms, including encouraging better communication between different providers when patients feel they are getting conflicting messages, suggesting that goals of care be revisited as care trajectories evolve, or simply requesting additional spiritual, religious, or other methods of support.

## Barriers to integrating SPC

Barriers to SPC integration generally present in settings where the benefits of SPC aren’t appreciated. Perhaps most prevalent is the misconception that palliative care is synonymous with hospice. In many instances, the ideal window to practice SPC is well before considering transition to hospice. If the differences between these two philosophies are not clear, patients and surgeons may hesitate to involve palliative care specialists [5]. This confusion can be avoided by educating both patients and clinicians about the application and benefits of palliative care for patients who are not dying. It may be helpful to clarify that palliative care can address quality of life for any patient and is not limited to those who are transitioning to comfort-focused care [12].

Funding is also a barrier for SPC, for both clinical work and research [8]. Palliative care as a whole is underfunded due to low reimbursement by healthcare systems, which leaves hospitals to absorb as much as 50% of costs related to palliative services [13]. As SPC continues to grow, this difficulty with reimbursement remains. In addition, there is a prevalent perception among surgeons that formal training in palliative care will not increase their earning potential and may even reduce it. Palliative care practice generates less revenue per unit time than surgery and this discrepancy may be reflected in compensation [14].

## Conclusions

Palliative care is inherently part of surgical care. In addition to seeking cures for pathology, every member of the surgical team must be mindful of how their treatment reflects what is most important in their patients’ lives. Surgeons, nurses, trainees, and other team members must recognize and take advantage of the opportunities to practice palliative care principles that present themselves naturally. These moments may be turning points in the way your patients relate to their medical providers and to healthcare as a whole. Our goal as surgical providers must be to empower patients, families, caregivers, and all members of the healthcare team to constantly assess and voice concern about the alignment of our treatment approaches with what our patients value most. As our healthcare delivery continues to evolve, integrating these principles will no longer set certain providers apart but will become the expectation for each and every member of the surgical team.
